# Conservative care with or without manipulative therapy in the management of back and neck pain in Danish children aged 9–15. Study protocol for a randomized controlled trial

**DOI:** 10.1186/s12998-016-0086-y

**Published:** 2016-01-28

**Authors:** Kristina Boe Dissing, Jan Hartvigsen, Niels Wedderkopp, Lise Hestbæk

**Affiliations:** Department of Sports Science and Clinical Biomechanics, Faculty of Health Sciences, University of Southern Denmark, Campusvej 55, DK-5230 Odense M, Denmark; Nordic Institute of Chiropractic and Clinical Biomechanics, Campusvej 55, DK-5230 Odense M, Denmark; Institute of Regional Health Services Research, University of Southern Denmark, Winsloewparken 193, DK-5000 Odense C, Denmark; Orthopaedic Department Hospital of Lillebaelt, Sports Medicine Clinic, Østre Hougvej 55, DK-5500 Middelfart, Denmark

**Keywords:** Randomized controlled trial, Children, Adolescents, Spinal pain, Manipulative therapy, Neck pain, Back pain

## Abstract

**Background:**

Complaints in the musculoskeletal system often start early in life and back and neck pain in children are well-established predictors for similar problems in adulthood. Despite lack of evidence of effectiveness, manipulative therapy is one of the most commonly used treatment modalities for back and neck pain in children.

The primary objective of this study is to evaluate the effectiveness of manipulative therapy when added to an approach consisting of manual soft tissue treatment, exercises and advice as needed, in children aged 9–15 complaining of back and neck pain.

**Method:**

The project is nested in the Childhood Health, Activity and Motor Performance School Study, which includes around 1200 children aged 9–15, who were all invited to participate in this randomized controlled trial in case they experienced back and/or neck pain during the two year inclusion period. Parents received text messages (SMS) on a weekly basis inquiring about the child’s musculoskeletal pain. If pain was reported, the child was evaluated for inclusion into the trial and, if eligible, randomized into one of two intervention groups:

1. Pragmatic advice, manual soft tissue treatment and exercises
2. The above plus manipulative therapy

By the end of data collection 237 children were included in the study. The primary outcome measure is number of recurrences of back and neck pain during the follow-up period (3–27 months). Secondary outcome measures are average duration of complaint time for each episode, total duration of complaint time, global perceived effect after two weeks, and change in pain intensity after 2 weeks. Baseline information includes quality of life, expectations to treatment, expectations to future course, age, gender, social class and physical education at school.

**Discussion:**

For most common non-traumatic musculoskeletal complaints no standardized and evidence based treatment strategy exists. We want to evaluate the effectiveness of manipulative therapy in addition to an approach consisting of manual soft tissue treatment, exercises and advice as needed, in children aged 9–15 complaining of back and neck pain.

To our knowledge this is the first large scale randomized controlled trial investigating the effectiveness of commonly used treatments for back and neck pain in children.

**Trial registration:**

ClinicalTrials NCT01504698

**Electronic supplementary material:**

The online version of this article (doi:10.1186/s12998-016-0086-y) contains supplementary material, which is available to authorized users.

## Background

Complaints in the musculoskeletal system often start during childhood and adolescence [1–4], and back and neck pain in children and young people are well-established predictors for similar problems in adulthood [5–8]. Besides the complaints directly related to pain or reduced mobility, these problems can also be a barrier to children’s physical activities, which may influence both physical and psychological health [9, 10]. Therefore, limitations caused by musculoskeletal pain in childhood can lead to musculoskeletal problems as well as potentially other lifestyle diseases like diabetes or cardiovascular diseases in adult life [11].

Low back pain is the most important of the musculoskeletal complaints from a socioeconomic perspective and is now ranked as the leading cause of years lived with disability in the world while neck pain is ranked fourth [12]. Back and neck pain has also been shown to be common in children, but for many children, the pain is mild in nature and of low intensity [13, 14]. However, some children are more severely affected, and this group is of particular interest in terms of prevention and treatment. Furthermore, it has been shown, that back and neck pain in children may progress; both to more locations in the spine, to higher frequency of pain, and to a higher pain intensity [13].

Thus, a focused effort directed towards early effective treatment of musculoskeletal problems in childhood to reduce recurrences, i.e. secondary prevention, appears justified. In fact this may be necessary if we want to maintain physical activity and limit long-term weakness and reduced function in the population caused by back and neck pain and other musculoskeletal disorders.

A positive effect of manipulative therapy (MT) in adults with various musculoskeletal problems is well-documented [15–18], e.g. for low back pain, where the effect is equally as good or better than usual care [18], and for several extremity joint conditions too [15, 19]. However, the evidence of effect in children is very sparse [20–23] and none of the studies relate to spinal pain. The choice of using MT on children can therefore only be based on tradition as well as on indirect evidence from trials and clinical guidelines for adults. The implications of using untested treatments on children are uncertain. Since they may not respond similarly to adults, they may require different dosages and experience different frequencies of side effects. Presently, MT is the most frequently used treatment of musculoskeletal complaints in children [24, 25], and in Denmark alone chiropractors treat around 17,000 children under the age of 18 every year, with musculoskeletal complaints being the most common one [10]. Therefore, it is of absolute importance to investigate the effect of this commonly used treatment strategy, which is actually considered to be best practice at the moment, despite lack of scientific evidence [21, 24, 25].

The purpose of this paper is to describe the methodology of a randomized controlled trial examining the effectiveness of MT when added to an approach consisting of manual soft tissue treatment, exercises and advice as needed, in children aged 9–15 complaining of back and neck pain. We hypothesize that the addition of manipulative therapy will decrease the risk of future episodes as well as the duration of episodes.

## Method

### Study design

#### Randomized controlled trial

##### Participants and setting

The project is a sub-study of The Childhood Health, Activity and Motor Performance School Study (CHAMPS). The CHAMPS study is a longitudinal cohort study that includes app. 1200 children aged 9–15 from 13 primary schools in the municipality of Svendborg, which is considered to be representative of the Danish population [26]. The main purpose of the overarching study is to evaluate the influence of extra physical education on the amount of musculoskeletal injuries and on childhood health in general. The schools were divided into two groups: one receiving the normal amount of two physical education lessons per week and the other one receiving six lessons per week.

The CHAMPS study started in 2008 and the data collection on injuries and back problems ended in summer 2014. The research team consisted of researchers with a range of professional backgrounds and from different departments all investigating different aspects of childhood health. At baseline, the children and their parents filled out a questionnaire addressing age, gender, health status, social class, work and leisure time activities. Social class was derived from parental educational level. The children have been followed with different kinds of testing throughout the study, e.g. physical tests, blood samples, DEXA scans, and, most importantly, three weekly text messages (SMS) sent to their parents inquiring about the child’s musculoskeletal complaints and the amount and type of leisure time sports activity during the past week (Additional file 1: Appendix 1). Parents answered using the reply function, and these were automatically registered and stored in a database. If they did not reply, they automatically got a SMS reminder two times during the following week. The SMS-response is a very efficient way to obtain frequent information and has been proven effective [27], and the response rate has been above 92 % in the CHAMPS study.

When a parent responded that the child had experienced pain during the previous week, a member of a screening team, consisting of three chiropractors and two physiotherapists, phoned the parents and administered a standardized interview regarding the complaint. Based on this, the interviewer determined whether the complaint was negligible or whether the child should be seen by a member of a clinical team that consists of five chiropractors with at least 3 years of clinical experience. The decision was made from anamnestic information about the history of the complaint, the duration and possible cause of complaint, the nature of the pain and if the pain seemed to be self-limiting or of a more prolonged nature. The examination took place at the child’s school, and following the examination the child received a diagnosis if possible, and was offered advice on how to handle his or her problem too. The same information was given to the parents either by phone or letter.

### RCT

#### Recruitment

**Fig. 1:**
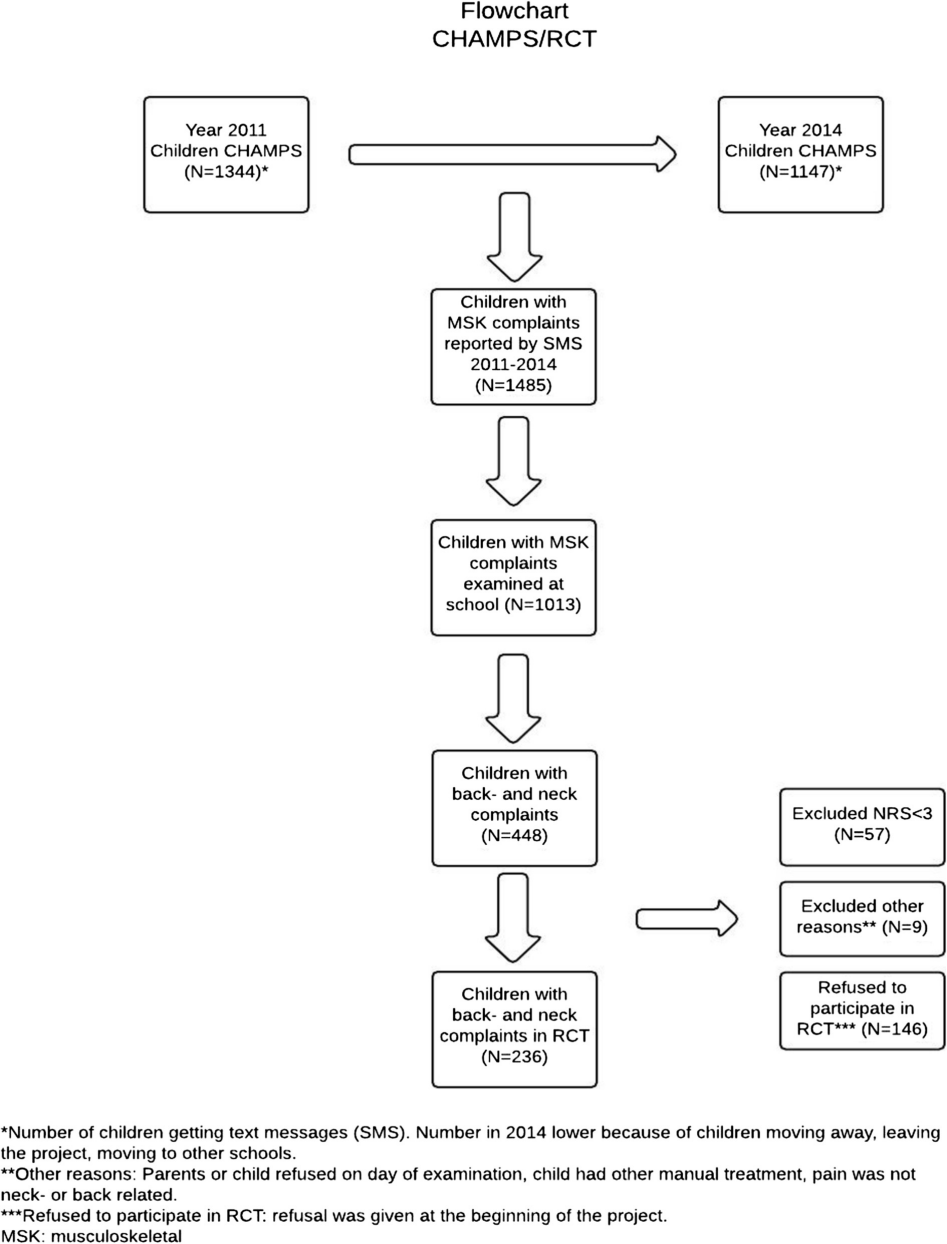
Flowchart CHAMPS/RCT

In 2012, all enrolled children (see Fig. 1) were invited to join this randomized controlled trial if they experienced back and neck pain during the study period (2 years), i.e. they accepted participation pending a future episode of back and neck pain. Children not enrolled and new coming children had the possibility to join the study throughout the study period. There was a start-up period from February to March 2012 where procedures and logistics were tested as well as the feasibility of the self-reported outcome measures, i.e. the NRS scale and the KIDDS screen questionnaires. Because no problems were encountered and no alterations were made, the trial continued unaltered. The children were followed until the end of school in the summer of 2014.

### Ethics

Temporary reddening and soreness in the treated area is common after both soft-tissue and manipulative treatment. No serious or lasting side effects have ever been reported in children aged 9–12 following the types of treatment used in this trial and no compensation claims have ever been made for this age group in Denmark [28]. Because there is no experimental treatment involved, but only treatments, which are usually performed in clinical practice, no interim analyses were made.

All parents have given written informed consent for their child to participate in the study. Participation in this trial is voluntary and the parents could withdraw their child from the study at any time with no negative consequences for the child. All participants were treated according to the Helsinki declaration [29].

The project has been approved by The Regional Committee on Health Research Ethics (#S-20110042) and data are being handled according to regulations by the Danish Data Protection Agency (#2013-41-1738).

#### Procedure

If a parent answered positively for back and neck pain on the weekly SMS and the telephone interviewer found that the child possibly was eligible for the trial, a member of the clinical team would evaluate the child at his or her school for inclusion or exclusion criteria (see Table 1).

**Table 1:** Inclusion and exclusion criteria

Inclusion criteria	Exclusion criteria
Pain in neck or back equal to or greater than 3 on an 11-box numerical rating scale for more than three days	Serious pathology (cancer, inflammatory diseases, vertebral fractures, cauda equina)
	Manual treatment for the past 2 months (for this particular complaint)
	Handicaps preventing normal physical activity
	Contraindications to manipulative therapy

**Fig. 2:**
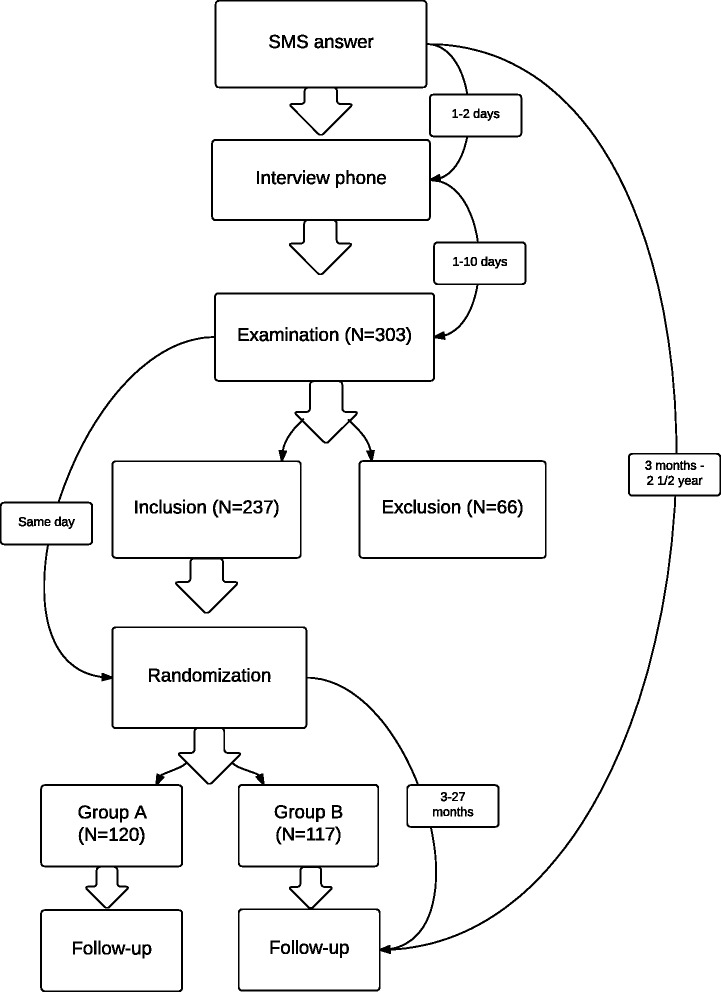
Flowchart RCT

At the first visit, the chiropractor took down a thorough history that included the rating of pain on a numerical 11-box rating scale. If the child fulfilled the inclusion criteria of NRS (3 or more on a numerical rating scale) [30, 31], he or she was randomized to treatment in either group A or B (see Fig. 2).

At baseline, the children filled in the KIDDS screen questionnaire that is a quality of life measure specifically designed for children [32] and answered a question about their expectations to the course of their treatment. In addition, they underwent an objective clinical examination including relevant neurologic and orthopedic examination as well as general and segmental movement palpation of the spine. General movement palpation is defined by the practitioner moving the spine in all directions and noticing the potential lack of movement, e.g. diminished forward bending of the neck. They then received a working diagnosis and were treated according to the randomization group. If the children did not fulfill the inclusion criteria, they were advised to remain active, and if necessary they were referred to examination and/or treatment elsewhere. If a child enrolled in the study experienced a recurrence of the original complaint or a new complaint during the remaining project period, the whole procedure was repeated starting with the phone interview and judgment of severity as defined previously. The only exception was randomization, as the child stayed in the original randomization group throughout the whole study period regardless of the number of recurrences or new complaints (incl. complaints in the extremities).

All clinical information was filed in a web-based register (Clinic Care Web), the KIDDS screen questionnaire, was paper-based and entered manually into Epidata, and data from the SMS were automatically stored in a secure database. Back up of all data were stored on a secure server at the University of Southern Denmark.

Data was monitored by an employed data manager throughout the project period.

### Randomization

A research assistant, not otherwise associated with the study, performed a computer generated block randomization with block sizes randomly changing between 2 and 6 at the time of inclusion using a 1:1 allocation to one of two intervention groups A or B. He then wrote the consecutive letters of the two groups on separate pieces of paper and placed them in sealed opaque envelopes. These were given to the treating chiropractors. The intervention group was not revealed to the child or parents.

### Interventions

#### The non-manipulative group received

- Pragmatic advice such as the use of cold or hot packs, braces, taping, suitable activities, ergonomics etc.
- Exercises including self-stretching and/or strengthening exercises
- Soft tissue treatment in the form of manual trigger point therapy and/or massage. Assisted stretching was not allowed in this group, as this would approach mobilization

#### The manipulative group received

- The items mentioned above
- Manipulative therapy: joint manipulation consisting of high-velocity, low-amplitude manipulation and/or joint mobilization without a high-velocity impulse to the spine and/or the extremities where indicated based on movement restriction and/or pain response during movement palpation

Thus, manipulative therapy was administered when there was a perceived biomechanical dysfunction of one or more joints that the treating clinician related to the child’s symptoms. The purpose of MT is to eliminate or relieve the pain as well as to reestablish better mobility and enhance the biomechanics of the joint, thus creating a basis for normalization of muscle activity around the joint [33–35].

In both groups, the frequency and content of treatments was determined on a pragmatic basis by the treating chiropractor. The treatment was intended to resemble pragmatic daily clinical practice in order to make the results more generalizable and implementable. The treatment continued until cessation of symptoms as determined by the child or parent or until the treating chiropractor decided that no further treatment was warranted. After 2 weeks of treatment, or earlier if the treatment was terminated, the child was questioned about global perceived effect, NRS and satisfaction with treatment. If there was no improvement in symptoms after 4–6 weeks of treatment, the child was referred to a secondary care spine center for a second opinion and further diagnostic work-up and/or imaging. The child and/or parents could stop the treatment at any time and still participate in other parts of the CHAMPS study.

### Blinding

The interventions used in this trial make blinding of care providers impossible. The children were somewhat blinded because they were not told which group they were allocated to and the two groups would more or less have the same amount of treatment in terms of number of visits and time spent per visit. However, concealment of treatment group was difficult and some children might have detected the difference between the groups by comparing with their friends or by talking to their parents; or some may have had manipulative therapy before.

The parents filled in the weekly SMS-track at home independent of clinicians or researchers. For the analyses, the coding of treatment groups will be unknown to the primary investigator (KBD) and the statisticians performing the analyses, and the primary investigator is not involved in the treatments. The code will not be broken until the analyses are completed.

### Outcome measures

#### Primary outcome measure

Number of recurrences during the follow-up period (3–27 months). A recurrence was defined as: i) a positive answer for back and/or neck pain on the weekly SMS question “*Has [name of child] had any pain during the past week?”*; ii) at least one pain free week prior to the recurrence; iii) pain location in the same region as initial episode.

Back and neck pain was defined as three spinal regions: cervical pain, thoracic pain and lumbopelvic pain. The reason for combining lumbar and pelvic pain is that prior experience in the study showed that children often tended to define pelvic pain as lumbar pain and did not differentiate between the two.

#### Secondary outcome measures

- The average complaint time for each episode (measured in weeks).
- Information on pain site was collected from interviews and examinations and subsequently from the SMS-track. The number of recurrences and complaint time was collected by using data from the SMS-track (Additional file 1: appendix 1).
- Total duration of complaint time (measured in weeks). This was extracted from the SMS data (continuous variables).
- Global perceived effect after two weeks. The child was asked: how will you describe your general wellbeing now in your neck/back (and any extremities) as opposed to 2 weeks ago before treatment was started? This was rated on a 7-point Likert scale with 1 being much better and 7 being much worse.
- Change in pain intensity after two weeks. This was rated on an 11-point numerical rating scale where 0 is no pain and 10 is worst pain (continuous variable).

Finally, any side effects to the treatment were recorded at each clinical visit, if reported by the child.

In addition the following information was collected at baseline for descriptive purposes:

- Quality of life (KIDDS screen) (At baseline and at recurrent or new episodes).
- Expectations to treatment. The child was asked prior to treatment: how do you expect the course of your problem will be? This was rated on a 5-point scale with 1 being much worse and 5 being much better.
- Expectations to future course: if your problem goes away, do you expect it to recur? Answer: yes/no. (At baseline and at recurrent or new episodes).

Age (9–15 year), gender (boy, girl), educational level (1 = No qualification, 2 = Vocational training, 3 = Higher education < 3 years, 4 = Higher education 3–4 years, 5 = Higher education >4 years), intervention group (A, B), school (11 schools), grade (4^th^ to 9^th^ grade), physical education at school (extra physical education, normal physical education).

### Power considerations

The power of this study does not only depend on the treatment effects, but also on the average values of the primary outcomes and their inter-individual variation. To obtain a realistic judgment of the power of the study, a formal power calculation was postponed until the data collection was finished. Only information from each child regarding spinal pain or not for each week, and its school and class membership was used for the power calculation. Actually, we used this data to determine the power of the analyses for the primary outcome and the two outcomes based on the weekly SMS data in a small simulation study. In each simulation step we split the children randomly into two groups and removed randomly 20 % of all episodes in the simulated manipulative group, and shortened 50 % of all episodes of two or more weeks duration by 50 %. In this scenario, we observed a power of 76 % for the primary outcome (number of recurrences), of 20 % for the average length and of 87 % for the overall complaint time.

The lack of power for the average length is due to the fact that more than 40 % of all episodes have a length of one week. Removal of these short episodes results in an increase of the average length, counterbalancing the shortening of long episodes.

### Statistical analyses

The primary outcome of the study is the number of recurrences in a child.

The definition and analysis of this outcome is based on the following considerations:

For each weekly SMS sent after randomization a child is regarded as being affected by the original complaint, i.e. experiencing a recurrence, if there is a positive answer to the question "Has … had any pain during the last week?" and if the pain is located in the same region. The child is regarded as experiencing a recurrence, if the child was unaffected the previous X weeks (with X ≥ 1 in the main analysis and X ≥ 3 in later sensitivity analyses). The corresponding time at risk for a recurrence is the number of weeks the child is not affected prior to the recurrence. The treatment effect on the number of recurrences is assessed by a hierarchical negative binomial regression model with the number of recurrences as outcome and the time at risk as exposure time variable. School and classes will enter as random effects. Robust standard errors will be used to take a violation of the distributional assumption into account. Intervention effects will be expressed as incidence rate ratio.

Secondary outcomes:

1. Average length of an episode
2. Average complaint time

The definition and analyses of these two outcomes are based on the following considerations:

An episode starts directly after randomization and with each new recurrence there starts a new episode. The length of an episode is the number of consecutive weeks where the child is affected in the same region. For the episode starting with the randomization, one additional week prior to randomization is assumed. The treatment effect on the average length of episode is analyzed by using a hierarchical linear model with the length of each episode as outcome, the treatment indicator as covariate and school, class and subject as random effects. If the child is affected at the end of the follow up period, this (censored) episode is not included in the analysis. Interventions effects will be expressed as the difference in mean length. Since more than 40 % of all episodes have a length of more than one week, we will also compare the histograms of the length of episodes between the two groups to get a better understanding of the effect on the length of the episodes.

The overall complaint time is the number of weeks a child is affected. The treatment effect on the overall complaint time will be analyzed using a hierarchical negative binomial regression model with the overall complaint time as outcome and the time in study as exposure time variable. School and classes will enter as random effects. Robust standard errors will be used to take a violation of the distributional assumption into account. Intervention effects will be expressed as incidence ratios, which correspond here to ratios of the average complaint time per year.

Two further secondary outcomes:

- 1. Global perceived effect
  This outcome on a 7-point scale will be analyzed using a hierarchical linear model with the treatment indicator as covariate and school and class as random effects. Robust standard errors will be used to take the violation of the distributional assumption into account. Treatment effects will be expressed as difference in mean perception.
- 2. Change in pain intensity
  This will be analyzed in the same manner as the global perceived effect. Treatment effects will be expressed as difference in mean change.
  All analyses will be repeated separately for cervical complaints, thoracic complaints and lumbopelvic complaints. For all analyses, the covariates quality of life, expectations to treatment, expectations to future course, age, gender, social class, intervention group and physical education at school will be included in the models where relevant.
  A cluster effect of school and class will be taken into account using STATAs cluster option in all analyses.
  A sensitivity analysis will be made looking at number of pain free weeks prior to a recurrent or new event; will there be any difference if the pain free period changes from 1 week to 3 weeks.
  Significance level will be set to 5 %
  All results will be published in relevant peer reviewed scientific journals.

## Discussion

Severe traumatic musculoskeletal injuries in children are treated in the emergency department by a specific treatment strategy, but for most common non-traumatic musculoskeletal complaints no standardized and evidence based treatment strategy exists. To our knowledge, this is the first randomized controlled trial investigating the effect of MT on children complaining of back and neck pain. This is important due to the potential long-term consequences of musculoskeletal complaints in children and the lack of evidence based treatments. It is necessary to focus research efforts on how to best treat and prevent these complaints at an early age.

Many adults experience complaints in more than one region of the spine and therefore it is increasingly common to investigate the effect of manipulative therapy on complaints involving the whole spine rather than region-specific complaints [36, 37]. Symptoms from the various regions are very similar [38, 39], and pain in different regions of the spine may be closely interrelated. Furthermore, new research have shown that in children pain is likely to progress to more locations [13]. Therefore it is an important aspect of this study that the spine is treated both as one entity and as three separate regions.

The strengths of this study are that it is school based and nested in a large longitudinal cohort study where the children were monitored every week for two and a half years, and the pragmatic design makes the interventions easy to implement in daily practice. During the study period both groups received optimal pragmatic usual care with MT as the only difference. Therefore any difference in the results obtained between the two groups can be attributed to MT alone. For ethical reasons, we did not have a control group receiving no treatment, and we did not compare with “real life” usual care, which often is probably less than our pragmatic usual care. Because of the pragmatic setup, we did not have standardization on number or duration of treatment. However, in the analyses, we will determine if the number of visits differed between groups and if that is the case, the number of visits will be included in the explanatory models.

Blinding of the children and the practitioners was not possible due to the nature of the treatment. The results might be influenced by the interaction between the children and the practitioners; that includes verbal communication, physical contact and empathy between the two parts. These non-specific factors cannot be measured and we do not know the full influence of them in this trial. All children were however treated by more than one clinician, which will enhance generalizability, and choice of treatment in the individual consultation depended on the treating chiropractor.

A limitation of the study is, that we did not systematically ask for side effects to the treatment; it was only recorded if told by the child or if the practitioner occasionally asked for it. A systematic recording of side effects should be implemented in future studies

If it is possible to develop efficient treatment for back and neck pain in children and adolescents, a life course of recurring problems may be altered with potential positive implications for both individuals and society. And because it is very rare to have serious side effects to manipulative therapy in children, potentially just mild side effects as soreness or reddening [40], the possible implications in terms of improved spinal health and wellbeing may be considerable.

Furthermore, fast and complete recovery from back and neck pain will minimize the restrictive impact of the pain on the level of physical activity and thus potentially have a positive influence on general health. This is exceedingly important in this age group where the level of physical activity tend to decrease [41–43], which might have a significant impact on future health [44, 45], and where lifetime habits are being developed [43, 46].

### Trial status

Patient recruitment ended in summer 2014.

#### Trial registration

ClinicalTrials NCT01504698

## Additional file

Additonal file 1:
**Appendix1.** (DOCX 40 kb)

## Abbreviations

MT: Manipulative therapy
CHAMPS: The Childhood Health, Activity and Motor Performance School Study
SMS: Text messages
