# Supplementary material for: Conservative care with or without manipulative therapy in the management of back and neck pain in Danish children aged 9–15. Study protocol for a randomized controlled trial
Source: Chiropr Man Therap. 2016 Jan 28;24:5. doi: 10.1186/s12998-016-0086-y (PMC4730742; doi:10.1186/s12998-016-0086-y)
Supplement: Additonal file 1: — Appendix1. (DOCX 40 kb) [file 12998_2016_86_MOESM1_ESM.docx]

**Appendix 1**

Has <FIRSTNAME> had pain for the last week?

1.Neck, back or lumbar spine

2.Shoulder, arm or hand

3.Hip, leg or foot

4.No, my child has not had any pain

How many times has <FIRSTNAME> been to organised sports in his/her leisure time in the past week?

0 = 0 times

1 = 1

2 = 2

3 = 3

4 = 4

5 = 5

6 = 6

7 = 7

8 = more than 7 times

<FIRSTNAME> which kinds of sports?

1 Soccer

2 Handball

3 Basketball

4 Volleyball

5 Gymnastics

6 Tumbling

7 Svimming

8 Horse back riding

9 Dancing

10 Other
